# Study of the Efficacy of Artificial Intelligence Algorithm-Based Analysis of the Functional and Anatomical Improvement in Polynucleotide Treatment in Knee Osteoarthritis Patients: A Prospective Case Series

**DOI:** 10.3390/jcm11102845

**Published:** 2022-05-18

**Authors:** Ji Yoon Jang, Ji Hyun Kim, Min Woo Kim, Sung Hoon Kim, Sang Yeol Yong

**Affiliations:** 1 Department of Rehabilitation Medicine, Yonsei University Wonju College of Medicine, Wonju 26426, Korea; raichu11@naver.com (J.Y.J.); rehjhkim@yonsei.ac.kr (J.H.K.); kme4221@hanmail.net (M.W.K.); 2 Yonsei Institute of Sports Science and Exercise Medicine, Wonju 26426, Korea

**Keywords:** knee osteoarthritis, texture analysis, polynucleotide sodium, artificial intelligence algorithm

## Abstract

Knee osteoarthritis (OA) is one of the most common degenerative diseases in old age. Recent studies have suggested new treatment approaches dealing with subchondral remodeling, which is a typical feature of OA progression. However, diagnostic tools or therapeutic approaches related to such a process are still being researched. The automated artificial intelligence (AI) algorithm-based texture analysis is a new method used for OA-progression detection. We designed a prospective case series study to examine the efficacy of the AI algorithm-based texture analysis in detecting the restoration of the subchondral remodeling process, which is expected to follow therapeutic intervention. In this study, we used polynucleotide (PN) filler injections as the therapeutic modality and the treatment outcome was verified by symptom improvement, as well as by the induction of subchondral microstructural changes. We used AI algorithm-based texture analysis to observe these changes in the subchondral bone with the bone structure value (BSV). A total of 51 participants diagnosed with knee OA were enrolled in this study. Intra-articular PN filler (HP cell Vitaran J) injections were administered once a week and five times in total. Knee X-rays and texture analyses with BSVs were performed during the screening visit and the last visit three months after screening. The Visual Analogue Scale (VAS) and Korean-Western Ontario MacMaster (K-WOMAC) measurements were used at the screening visit, the fifth intra-articular injection visit, and the last visit. The VAS and K-WOMAC scores decreased after PN treatment and lasted for three months after the final injection. The BSV changed in the middle and deep layers of tibial bone after PN injection. This result could imply that there were microstructural changes in the subchondral bone after PN treatment, and that this change could be detected using the AI algorithm-based texture analysis. In conclusion, the AI- algorithm-based texture analysis could be a promising tool for detecting and assessing the therapeutic outcome in knee OA.

## 1. Introduction

Knee osteoarthritis (OA) is a musculoskeletal disorder affecting approximately 10% of adults aged over 55 years old. A previous study reported that approximately 12% of the aging population in the West suffer from knee OA, and 25% of the population above 55 years of age have experienced a persistent knee pain episode [1]. According to another study on the alignment and range of motion of the knee [2], in the elderly, many complain of clinical features of osteoarthritis, but findings pertaining to knee arthritis on X-rays are not clear. Knee OA is caused by an inflammation of the synovium surrounding the knee joint, causing cartilage degeneration, and consequently leads to a subchondral bone restructuring process. Nowadays, OA is projected to become the fourth leading cause of disability [3]. Due to related pain, decreased physical activity, and, consequently, increased body weight, there is an increased burden on the knee, thereby causing a viscous cycle. Once damaged, there are limited treatment modalities to regenerate knee cartilage, so if physical activity decreases due to the pain, the synovial fluid also decreases, causing more stiffness in the knee [4]. As the amount of cartilage surrounding the knee bone decreases, the nociceptive nerves become stimulated through the nociceptors, causing pain in the joint [5].

Knee OA is characterized by changes in the subchondral bone, such as subchondral sclerosis or osteophyte formation. Therefore, OA is often diagnosed by findings with radiologic modalities (X-rays, CT, or MRI) using diagnostic tools such as Kellgren–Lawrence (K–L) classification [6]. Subchondral bone sclerosis is widely considered to be a hallmark of OA. In OA, despite the increase in bone volume fraction, the subchondral bone is hypo-mineralized due to abnormal bone remodeling. The sclerosis of periarticular mineralized tissues may be a biomechanical-compensation-based adaptation to widespread cysts and micro-damage in the subchondral bone, which render the subchondral bone structure more fragile [7]. Recent studies focused on this subchondral bone-remodeling process as an important pathophysiology of knee OA [8,9]. Due to an improvement in radiologic techniques, some studies using plain X-rays and MRIs have reported favorable outcomes when using a texture analysis as a diagnostic tool for OA, as well as when predicting progression [10,11,12]. The subchondral remodeling process leads to subchondral cyst formation, and these findings could result in changes in the bone density of subchondral structures, which could be detected with the texture analysis method [13]. Since automated texture analyses using a computed or artificial intelligence (AI) algorithm are emerging, recent studies have shown promising results and the possibility of its application as a diagnostic tool for OA, compared to conventional texture analysis methods [14,15,16]. With these findings in mind, we designed this study in order to verify the efficacy of the AI algorithm-based texture analysis in detecting microstructural changes in the subchondral tibial bones in X-rays of the knee, to determine whether it can be used as a tool for OA diagnosis.

The primary treatment approach of knee OA is non-surgical treatment [17]. Conservative therapies for knee OA include physical exercise and weight loss [18], intra-articular injections (such as PN, hyaluronic acid, and platelet-rich plasma) [19,20], knee bracing [21], physical modalities [22], and pharmacology [23], but their long-term effectiveness is limited in most therapeutic options; thus, knee replacement surgery is ultimately required for the majority of patients with knee OA [17,24]. A polynucleotide (PN), a polymer molecule generated by a covalent bond of nucleotides, forms a three-dimensional gel with high viscosity and elasticity, as a secondary structural reconstitution occurs due to its property of combining with large amounts of water following an intra-articular injection [25,26,27]. Studies have shown similar or even profoundly improved outcomes following intra-articular PN injections compared to hyaluronic acid (HA) injections [28,29]. Intra-articular PN injections reduce mechanical friction by serving as a buffer and lubricant through a cushioning effect, as well as the pain caused by mechanical friction by coating the synovium and thereby blocking the nociceptors within [26,29]. Additionally, in a retrospective study of a total of 60 patients (HA = 30 subjects; PN = 30 subjects) diagnosed with Class II knee arthritis for approximately six months, the results showed that both agents achieved statistically significant improvements in the Visual Analog Scale (VAS) and without any adverse events [26].

Based on previous studies, we expected that therapeutic interventions could restore the subchondral remodeling process. Intra-articular injections are the most widely used modality to treat pain in knee OA patients. Therefore, we designed a prospective case-series study using PN as the therapeutic modality. When PN fillers are injected intra-articularly, mechanical damage to the knee joint space and inflammatory damage to the subchondral structure are reduced. We focused on the mechanism of the PN filler and aimed to determine whether this treatment achieves not only functional improvement, but also whether it leads to the recovery of the micro-architecture of subchondral bone structures.

Our goal in this study was first to determine if PN filler treatment can achieve clinical outcomes not only in terms of symptomatic relief, but also with regard to disease-related improvements such as the restoration of subchondral remodeling. Second, our aim was to determine the efficacy of the AI algorithm-based texture analysis of the tibial bone in assessing treatment outcomes in knee OA patients.

## 2. Materials and Methods

### 2.1. Study Design

This prospective case series study was a single-center, observational study to verify the efficacy of the AI algorithm-based analysis in terms of functional and anatomical improvements following PN treatment in knee OA outpatients who were diagnosed with knee OA and suffered from pain and a limited range of motion. The subjects were recruited at Yonsei University Wonju Christian Hospital from 2019 to 2020. The enrolled subjects were treated with knee intra-articular injections of 2 mL of PN filler (HP cell Vitaran J, BRPHARM Co., Ltd., Wonju, Korea) at each visit for knee OA treatment, with a total of five injections per person.

### 2.2. Materials

The polynucleotide sodium used in this study (HP cell Vitaran J, BRPHARM Co., Ltd., South Korea) was provided in prefilled sterile syringes, containing 2 mL of colorless and clear-appearance solution. The product was released in 2018 in South Korea, a type of PN extracted from salmon. The solution has a 90–110% DNA content in a concentration of 20 mg/mL. The software program we used was the fully automated joint gap analysis method (the IB LAB Analyzer™, ImageBiopsy Lab, Vienna, Austria), which is a newly developed algorithm that examines the quality of the bone structure by solely using conventional X-ray images.

### 2.3. Study Population

Participants aged over 40 and under 80 years were enrolled in this study. Those that met the inclusion and exclusion criteria were enrolled in this study (Table A1), based upon their history and a physical examination. All participants voluntarily provided their written consent to participate after listening to an oral explanation of the study’s purpose. Those diagnosed with knee arthritis according to the ACR classification criteria, classified as Class I–III based on the K–L scale, as indicated by anterior–posterior plane radiographs, were selected as eligible subjects for the study. After obtaining consent, a screening test was performed to verify whether the subjects met the inclusion/exclusion criteria.

### 2.4. Experimental Intervention

All participants were given an intra-articular PN injection at an interval of one week, starting from the baseline visit (week 1) to the final injection visit (week 5), with a total of five injections per person. All injections were performed under ultrasound guidance with the superolateral approach. All clinical parameters of the outcome measurement were evaluated at each visit. Before being discharged from the study, each patient was asked to attend a follow-up visit 12 months after the final injection (week 16) to evaluate the short-term follow-up effects.

#### 2.4.1. Outcomes Measurements

The efficacy of PN-filler treatment in knee OA patients was evaluated in two different ways, by analyzing both the functional and the anatomical improvements of the micro-architecture of the tibial subchondral bone structure. Their functional improvement was evaluated using a Visual analogue scale (VAS) and Western Ontario and McMaster Universities (K-WOMAC) Osteoarthritis Index after PN injection. Anatomical improvement was evaluated by analyzing the BSV score of the subchondral bone region of interest (ROI).

The improvements in knee pain were assessed using VAS at resting (non-weight bearing) and weight bearing (single leg standing with knee flexion of 30 degree) positions. Participants were asked to report their VAS score of the knee during the weighted and non-weighted position by drawing a line perpendicular to the VAS at each visit of baseline, at the final 5th injection (week 4) and at the last follow-up visit (week 16). Changes in knee pain, stiffness, and physical function were assessed using the K-WOMAC Osteoarthritis Index. K-WOMAC was also surveyed at baseline, after the final injection, and at the last follow-up.

Plain radiographs were performed on both sides of knee for a bone texture analysis at the baseline visit and once more in the 16th week, which was three months after the last injection. In addition, a synovial biomarker test was performed during PN-filler treatment at visits two and six to assess improvements in the synovial inflammation (IL-6, IL-10, and TNF-α).

#### 2.4.2. Texture Analysis

**Figure 1:**
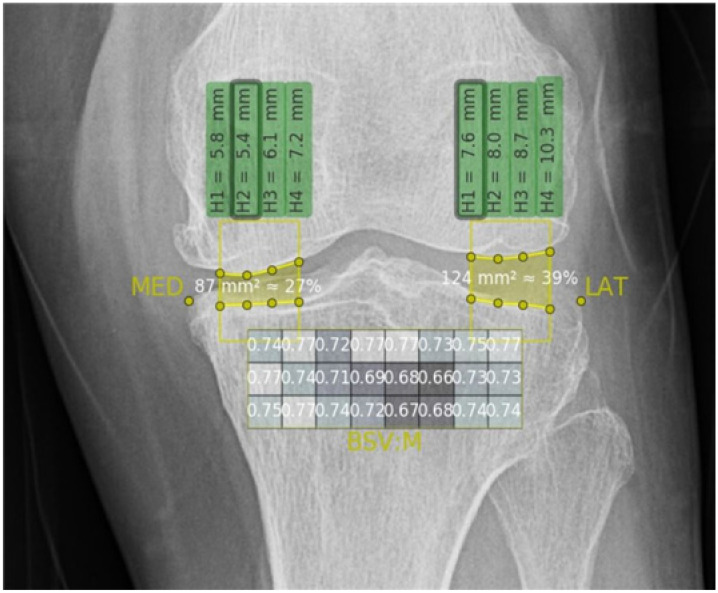
The 24 compartments divided by artificial intelligence software. The artificial intelligence software divided the trabecular bone into 24 compartments at the proximal diaphysis of the tibia in a knee X-ray, showing the BSV results of each compartment. BSV, bone structure value.

In this study, we used the fully automated AI algorithm model to analyze the changes in the upper tibial subchondral bone texture after intra-articular PN-injection treatment. Using a bone texture analysis, we were able to gain information on the subchondral structure status. We focused on whether the expected changes in the microstructure after the intervention could be detected with the texture analysis. The texture analysis was performed using plane radiographs of the tibial bone with AI algorithm software (the IB LAB Analyzer™, ImageBiopsy Lab, Vienna, Austria). This algorithm combines a set of advanced bone micro-architecture fractal algorithm parameters to offer a detailed assessment of the trabecular structure of the bone. Among the parameters, we used the bone structure value (BSV) to analyze changes in the microstructure of the tibial subchondral bone after PN-filler treatment. According to previous studies on radiocarpal and knee joints, BSV is considered as a maximum-likelihood estimator of OA [30,31,32]. The BSV algorithm analyzes the self-similarity (uniformity) in the fractal dimension of the bone support structures based on the gray values of an X-ray image. The BSV was measured automatically by the AI algorithm program in the subchondral tibia head area. It calculated how the gray values of a specified region of interest (ROI) was uniformly distributed. This algorithm can thus provide information about the smoothness and surface topography of the bone structure. It can also measure in the following directions: along the horizontal axis, the vertical axis, or the mean of them. A mask of 24 individual regions in a 3 × 8 grid in the subchondral tibia head is provided in Figure 1.

**Figure 2:**
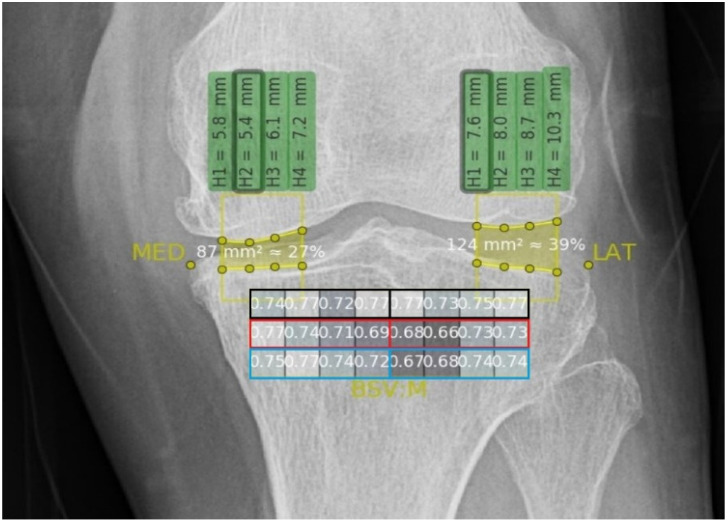
Subgroups of the 24 compartments according to their location and depth. Each layer is highlighted with different color borderlines: Black for the superficial layer, red for the middle layer, and blue for the deep layer. In addition, since each layer is divided into medial and lateral compartments, a total of 24 compartments were grouped into six subgroups.

Algorithm analyses of the BSV were obtained at each ROI using knee X-rays at baseline and at 16 weeks after the last injection. A previous study reported that there was a difference between OA progression in different compartments of the tibial subchondral bone (medial side more severe than the lateral side), with even more bone alternations in the deeper subchondral bone layers [10,33,34]. According to this study, the ROIs were divided into subgroups according to their location (medial or lateral compartment) and depth (superficial, middle, or deep layer) (Figure 2).

We focused on the previous study results, that highlighted a difference in the BSV between the medial and lateral compartments, and we aimed to investigate whether any alterations were observed in the BSVs of these compartments after PN-filler treatment in each layer. The knee X-ray of each participant was analyzed and classified into subgroups considering the pathology location of OA. We analyzed the BSV difference between the affected compartment and non-affected compartment of the knee. The delta BSV (ΔBSV) was defined as the affected compartment BSV—non-affected compartment BSV, and was analyzed before and after PN-filler treatment.

### 2.5. Rationale for Sample Size Determination

The sample size was estimated based on a decrease in changes in the pain and WOMAC scores. The effect size of the intra-articular visco-supplement therapy ranged from 0.60 to 0.65 according to previous studies [35,36]. Therefore, we calculated the sample size using G*Power 3 (RRID:SCR_013726) [37], a significance level of 5%, a statistical power of 95%, and a dropout rate of 20%, and we estimated a sample size of 50–56 to be suitable for this study.

### 2.6. Statistical Analysis

First, a one-way ANOVA was used to evaluate the therapeutic effects of the PN-filler treatment in our study participants. The VAS and K-WOMAC scores between baseline, the final injection (week 4), and the last follow-up (week 16) were analyzed. A one-way ANOVA was used to compare the BSV of medial and lateral compartment. A Tuckey’s test was conducted for post hoc analysis. Second, a Friedman test was used to compare the delta BSV, which is the difference between the affected compartments and non-affected compartments for each layer according to the OA site, and the *p*-values were adjusted using the Bonferroni multiple testing correction method. Third, a one-way ANOVA and Tuckey’s post hoc analysis were used to evaluate the changes between pre- and post-PN-filler injections in the ΔBSV (affected compartment BSV—non-affected compartment BSV). A *p*-value of ≤0.05 was considered statistically significant. All data were analyzed using SPSS version 25.0 (IBM Inc., New York, NY, USA), and R version 4.1.0 (https://www.r-project.org (20 May 2021)).

## 3. Results

**Figure 3:**
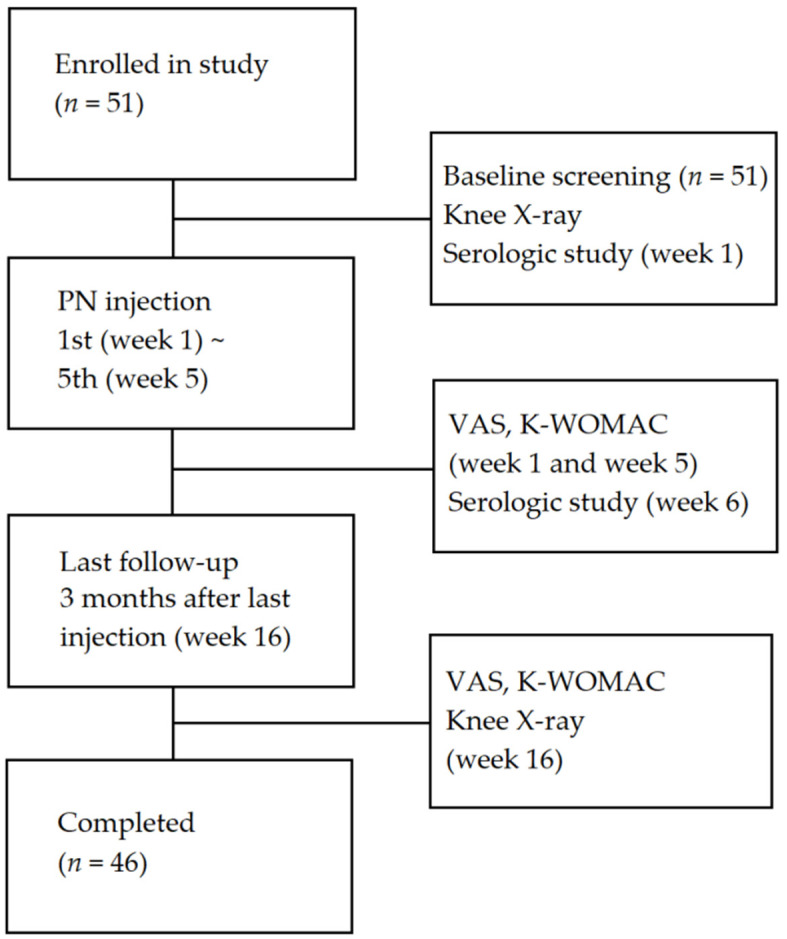
Diagram of the study protocol. Five participants dropped out (three participants did not attend the scheduled visit date, and two participants did not perform the scheduled X-ray examination).

A total of 51 participants were enrolled in this study. A total of five participants dropped out (three participants did not attend the scheduled visit date, and two participants visited but did not perform the scheduled X-ray examination), and were excluded from the analysis due to missing values (Figure 3). The demographic characteristics of the participants are listed in Table 1. There was a higher proportion of females (72.5%) than males (27.5%). The average age of the participants was 62.7 years, and K–L grade I (*n* = 28) was the most dominant, while K–L grade III (*n* = 4) was the least common.

### 3.1. VAS

**Figure 4:**
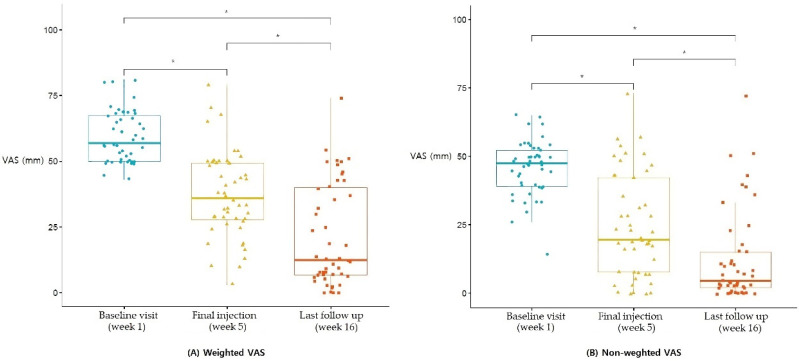
VAS score differences between the baseline, final PN injection, and last follow-up visit. (**A**) The VAS score (mean = 37.3 mm, SD = 16.4) of the weighted position was decreased by the time of the fifth and final PN injection visit compared to the baseline visit (mean = 59.3 mm, SD = 9.7), and further decreased by the last follow-up (mean = 21.5 mm, SD = 19.5) visit. (**B**) The VAS score (mean = 24.5 mm, SD = 18.9) of the non-weighted position decreased by the time of the fifth and final PN injection visit compared to the baseline visit (mean = 45.9 mm, SD = 10.1), and further decreased by the last follow-up visit (mean = 12.4 mm, SD = 16.8). The *p*-values are based on the results of a one-way ANOVA comparing the VAS scores among the groups; * *p* < 0.05. PN, polynucleotide; VAS, visual analogue scale.

The weighted VAS showed a statistically significant improvement after the fifth and final PN-filler injection compared to the baseline visit (*p* < 0.0001), and further improved at the last follow-up visit compared to the fifth and final PN injection (*p* < 0.0001). The non-weighted VAS also showed the same results. The non-weighted VAS of the participants showed a statistically significant improvement between the baseline and fifth and final injection (*p* < 0.0001), between the fifth and final injection and the last follow-up (*p* = 0.00068), and between the baseline and last follow-up visit (*p* < 0.0001) (Figure 4).

### 3.2. K-WOMAC

**Figure 5:**
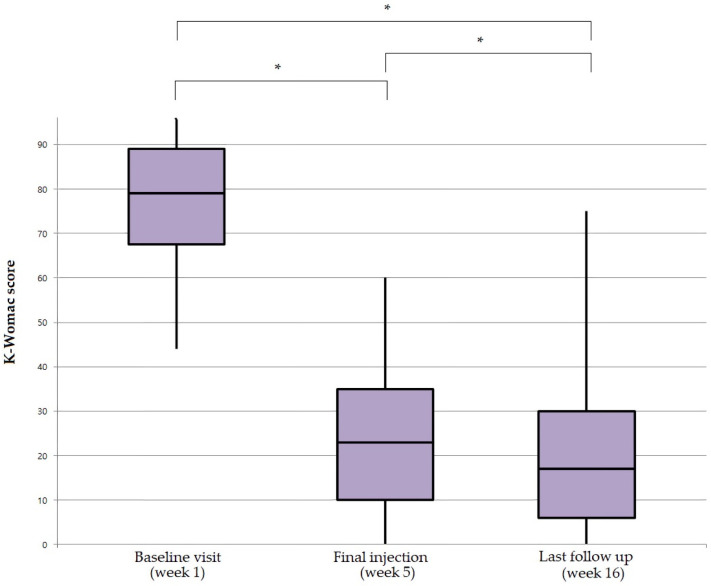
K-WOMAC score at baseline, final injection, and last follow-up visit. The K-WOMAC score decreased with time. At the time of the fifth and final injection, the K-WOMAC score (mean = 27.6) improved compared to the baseline visit (mean = 49.7), and further decreased by the last follow-up visit (mean = 25.3). The therapeutic effect lasted throughout the period after PN injection. The *p*-values are based on the results of Tuckey’s post hoc analysis after ANOVA for the comparison of K-WOMAC scores between the groups. K-WOMAC, Korean-Western Ontario MacMaster; PN, polynucleotide. * *p* < 0.05.

K-WOMAC was used to evaluate the pain, stiffness, and physical functions of the participants. The K-WOMAC scores showed statistically significant improvements after PN-filler injection by means of a decreased total K-WOMAC score compared to the baseline visit. The K-WOMAC score further decreased by the last follow-up visit (*p* < 0.012). The total K-WOMAC score decreased after the fifth and final injection, lasting until the final follow-up visit (Figure 5).

The pain score was taken as a sub score of K-WOMAC, and it showed improved outcomes after the fifth and final injection (*p* < 0.0001). Although the pain score did not further decrease by the last follow-up visit when compared to the fifth and final injection visit (*p* = 0.1), its therapeutic effect lasted until the final follow-up visit compared to the base line visit (*p* < 0.001).

The stiffness score showed significant improvement after the PN treatment (*p* < 0.0001) and last follow-up visit (*p* < 0.0001) compared to the baseline visit. It also showed significant improvement at the last follow-up visit compared to the fifth and final injection visit (*p* < 0.0001).

The physical function score also showed the same results, with a significant improvement between baseline and the fifth and final injection (*p* < 0.0001), between the fifth and final injection (*p* = 0.0093), and between baseline and the last follow-up visit (*p* < 0.0001).

### 3.3. BSVs

The BSVs were calculated using the automated algorithm with 24 ROIs mapped onto the tibial subchondral bone area (Figure 1). The 24 ROIs were divided into six subgroups based on their location (medial or lateral) and depth (superficial, middle, or deep layer) (Figure 2). The median BSV is provided according to the categorized subgroup (Table 2).

**Figure 6:**
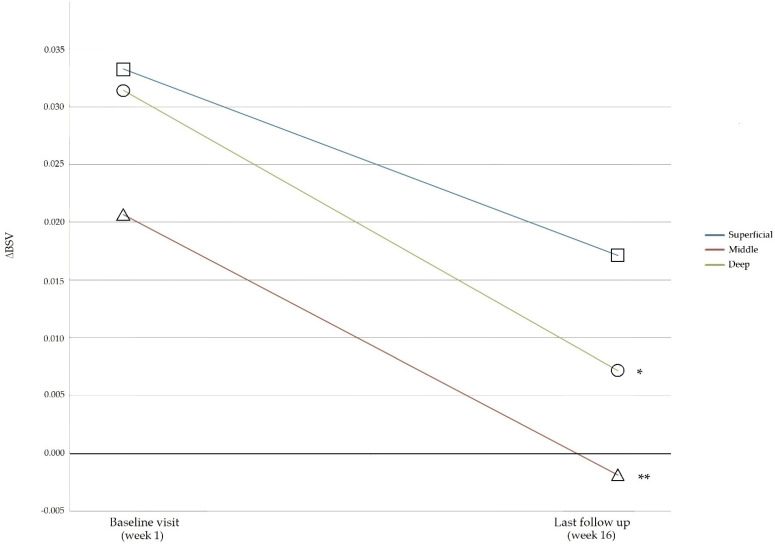
Delta BSV changes between baseline and the last follow-up visit in each sublayer. The delta BSV (affected compartment BSV-non-affected compartment BSV) did not show a statistically significant changes in the superficial layer, but decreased significantly in the middle and deep layers after PN injection. The *p*-values are based on the results of Tuckey’s post hoc analysis after ANOVA comparing the BSVs between the subgroups. * *p* < 0.001 and ** *p* < 0.005. BSV, bone structure value; PN, polynucleotide.

The median BSV was higher in all layers of the medial subgroup compared to the lateral subgroup. The mean BSV was highest in the superficial layer, while it was lowest in the deep layer. There were significant differences in the mean BSV between the medial and lateral groups for each layer (superficial, middle, and deep) before PN injection. Statistical significance between the medial and lateral subgroups disappeared after administration of the PN injection in the middle and deep layers, while statistical significance remained in the superficial layer. To re-examine this result, we analyzed the ΔBSV according to the OA-affected compartment side changes of each layer before (baseline visit) and after PN injection (last follow-up visit) (Table 3). The affected compartment was decided based on the patient demography of the OA site of the tibia (Table 1). The results showed no statistically significant differences between the sublayers at baseline and the last follow-up visit. The ΔBSV comparison between the baseline visit and the last follow-up visit showed no significance in the superficial layer. However, ΔBSV showed statistically significant changes between the baseline visit and the last follow-up visit in the middle (*p* < 0.005) and deep layers (*p* < 0.001). The *p*-values are based on the results of Tuckey’s post hoc analysis after ANOVA comparing the BSVs between the subgroups (Figure 6).

Serologic analysis showed no significant differences in the synovial inflammatory biomarkers (IL-6, IL-10, and TNF-α.) between baseline and the fifth and final injection visit.

## 4. Discussion

In this prospective case series, the aim was to determine the efficacy of the AI algorithm-based texture analysis in verifying the microstructural changes of the subchondral tibial bone after knee OA intervention. The results showed improvements in the VAS and K-WOMAC scores, as well as improvements in the BSVs in the middle and deep layers in the lateral compartment of the subchondral bone.

In our study, an improvement in the VAS and K-WOMAC scores in knee OA patients after the repeated treatment of intra-articular PN filler injections was verified, and the therapeutic effects lasted until a few months after the last injection. PN has a high molecular weight that can bind to a large amount of water. Thus, it is a substance that can increase in viscoelasticity, forming a three-dimensional (3D) gel-type matrix, which is more effective in preventing mechanical friction [38]. This mechanism leads to an improvement in physical symptoms (stiffness and physical functions) and decreased cartilage destruction, which can prevent subchondral remodeling aggravation. Moreover, PN undergoes anti-inflammatory interactions that can reduce the pain reaction induced in OA (nociceptive stimulation of pain signals, sensory nerve innervation, neuropathic sensitization, etc.) [39]. Based on these mechanisms, PN injections can produce functional improvements in knee OA patients.

A loss in the statistical significance of the differences in BSVs between the medial and lateral compartments in the middle and deep layers of the subchondral bone after PN filler treatment was also observed. These findings indicate that there may be improvements in the BSVs in the lateral compartment of the middle and deep layers of the subchondral bones after PN filler treatment. Additionally, a statistically significant difference of ΔBSV before and after PN filler treatment was noticed in the same layers. As the BSVs represent 3D fractals of the trabecular bone [40,41], improvements in the BSV in the middle and deep layers can lead to the conjecture that there is a possibility of radiographic fractal improvement, as well as a restoration of the micro-architecture of the subchondral bone structure.

However, there is not enough research on the theoretical background to validate these findings. The improvements in the BSVs could be an indication of the reversal of the subchondral bone remodeling sequence. A previous study reported that recovery from the subchondral bone remodeling process was observed after the treatment of ankle OA [42]. Other researchers reported that subchondral bone remodeling is reversible and that treatment of knee OA should focus on subchondral bone micro-environment regulation [8,9]. Regarding previous studies, we were able to come up with a few hypotheses about how BSV improvements could occur following PN treatment.

In response to aberrant mechanical loading and subsequent cartilage destruction, subchondral bone remodeling proceeds to a late stage with increased biological factors such as transforming growth factor (TGF)-β, vascular endothelial growth factor (VEGF), and matrix metalloproteinase (MMP) [9,43,44,45]. Recent studies detected TGF-β signaling in remodeling surrounding the bone matrix, by which osteocytes sense the mechanical alterations and resorb the surrounding bone matrix by producing MMPs, in which subchondral bone osteocytes can induce an OA-like phenotype, even in the non-injured knee, suggesting the important role of osteocytes in subchondral bone [46]. It is also known that MMP-2, -9, and -13 are deeply related in subchondral homeostasis [9,46]. Increased VEGF can also accelerate OA progression by recruiting osteoclasts and stimulating osteogenesis and angiogenesis [47]. This condition leads to cartilage erosion, increases MMP-9 expression, and leads to osteogenic defects and abnormally large growth plates, suggesting the critical role of endothelial-derived MMP-9 in cartilage resorption [9,45]. Therefore, previous studies suggested that the treatment of knee OA should target preventing such a sequence by regulating uncoupled bone remodeling (osteocyte and osteoblast regulation), regulating the inhibition of aberrant angiogenesis, or by encouraging the selective removal of senescent cells (drugs to block anti-apoptosis or cytokine-neutralizing antibody therapies) [8,9].

Polydeoxynucleotide (PDRN), which is also a DNA polymer extracted from the testis and semen of salmon or trout, respectively, is known to exert anti-inflammatory action without metabolic side effects. Previous studies have proven PN to have similar anti-inflammatory responses to PDRN when administered in vivo [48]. PDRN shows inhibitory effects on proteoglycan degradation in the cartilage and the activation of MMP-2 and MMP-9 in vitro [49]. It is also known to act as an adenosine A2A receptor [38,50]. As it works as an A2A antagonist, it exerts anti-inflammatory effects by inhibiting mast cell degranulation and inflammatory cytokine, thereby reducing pro-inflammatory mediators such as TNF-α, IL-6, and high-mobility group protein. Likewise, PN treatment results in an even greater decrease in the gene expression of IL-6, IL-1β, IL-8, and chemokine (C-C motif) ligand-3 genes compared to PDRN treatment [48]. Along with these study reports, PN injection can reduce inflammatory cytokine activation, thereby reducing cytokine-related cell injury, and can also inhibit MMP activity, which leads to subchondral remodeling. A recent study reported that blocking the PI3K/AKT signaling pathway can inhibit bone sclerosis and subchondral bone remodeling [51]. Since PI3K/AKT is regulated by the adenosine A3A receptor [52], the therapeutic effectiveness of PN may be functionally related to the adenosine A3A receptor, due to its structural similarity with PDRN, being an adenosine A2A receptor. The enzymatic degradation products of PN are released into the joint cavity, which physiologically present in the extracellular environment and are useful materials for cell regeneration via the regulation of enzymatic activities such as alkaline phosphatase [53,54]. PN degradation materials result in nucleotide, purine, and pyrimidine bases, which are used by cells to support the physiological process of repairing cartilage [55]. As purine and pyrimidine derivatives are known to be PI3K/AKT inhibitors [56], PN injections may play a role in inhibiting subchondral bone remodeling by suppressing the PI3K/AKT signaling pathway.

Subchondral bone remodeling is an emerging idea in the treatment of OA patients, but there is a lack of diagnostic tools for detecting the remodeling process or assessing treatment outcomes. The diagnosis of OA is based on assessing joint space width and is typically achieved by radiography. The K–L grade is the most commonly used classical diagnostic tool for OA. However, it has several limitations such as application to disease progression and insensitivity to change, or inconsistency in its original description by the authors and variable applications in classification in subsequent studies [57]. Moreover, it has limitations in assessing treatment effectiveness, which relies mainly on pain scores. Although the K–L system has been validated with respect to inter–intra-observer reliability and high diagnostic accuracy, researchers have suggested further diagnostic applications focusing on the classification grade related to or based on treatment algorithms due to its limitations [57]. Due to improvements in radiologic techniques, some studies using plain X-rays and MRIs have reported favorable outcomes when using texture analysis as a diagnostic tool for OA, as well as in the prediction of progression. Tibial subchondral bone texture analyses using plain X-rays have shown promising results in predicting the occurrence of knee OA and its progression [34,58]. Subchondral microstructural changes have been detected using MRI studies, also supporting that texture analyses with MRIs can also be good predictors of OA progression [13,59]. The automated texture analysis method provides numeric values, which can be used as a subjective diagnostic tool. Numeric values can easily provide information for disease progression. Finally, in this study, we verified that the AI algorithm-based texture analysis can detect microstructural changes in the subchondral bone in knee OA patients after therapeutic intervention. Therefore, the automated algorithm texture analysis method could be a promising tool for not only diagnosing and predicting OA progression, but also for assessing therapeutic outcomes in OA patients. We assume that the AI algorithm-based texture analysis method could be an alternative diagnostic method for the OA population, supplementing the limitations of classic diagnostic tools for OA such as the K–L grade.

### Limitations

The first and most important limitation of this study is that this was the first study designed to evaluate the therapeutic outcomes of using the texture analysis, but this was also the most meaningful part of this study. Since our study is the first study, it was difficult to find sufficient information to support the results of our study, so further research should be conducted to confirm the theoretical basis of the BSV analysis identified in our study and the hypothesis about the mechanism of PN influencing BSVs. In addition, our study was a prospective case series, thus lacking a control group. In future studies, it is absolutely necessary to define and design an appropriate control group. However, since previous randomized controlled trials (RCTs) showed good clinical results following PN injections in knee OA patients, we focused on whether the accompanying subchondral remodeling process could be confirmed with an AI algorithm rather than confirming the clinical effect after therapeutic intervention.

In this study, the lateral compartment of the middle and deep layers showed improvements in the BSVs. Additionally, our ΔBSV results showed greater BSV improvement in non-affected compartment. This result may indicate that the BSV of non-affected compartments showed more improvements compared to affected compartment. According to previous studies [2], despite a lack of arthritic features on X-rays, there may be underlying arthritis in knee OA patients. Therefore, the non-affected compartment may also undergo a subchondral remodeling process. The theoretical explanation of this result is not clear, but our research focused on the fact that the medial compartment is more likely to be involved in OA. By way of explaining our results, we considered that there was not enough of a therapeutic effect or not enough time for the microstructure of the medial compartment or the affected compartment to be resorted, showing fewer profound results in BSV changes. Another explanation could be the fact that PN treatment was carried out via a superolateral injection. After IA injection with the superolateral approach, the injected PN may remain more on the lateral side, and this could affect the remodeling process of the lateral compartment of the tibial subchondral bone. Moreover, the medial compartment of the tibial bone is known to be susceptible to OA. The participants in this study showed higher BSVs in the medial compartment than in the lateral compartment in this study. This result may be explained by the composition of the participants, as most of the participants had a low K–L grade, in which the medial compartment was not yet severely involved, resulting in higher BSVs. Finally, subchondral bone sclerosis arises in the superficial layers of the subchondral bone structure. Meanwhile, subchondral cyst formation in the bone remodeling process occurs in the deeper layers [7,8,9]. This can explain the results showing that the BSVs of the middle and deep layers were improved in our study.

Another limitation of our study is its hypothesis. PNs or PDRNs are known to enhance VEGF activity. In this way, PN injections should aggravate subchondral remodeling, but the results showed the opposite. Even though there are similar studies evaluating the efficacy of PN or PDRN, texture analyses for detecting subchondral remodeling reversal have never been researched. As texture analyses using an automated AI algorithm represent a newly arising field in diagnosing OA [15,16,33,58], further research should be conducted in this specific field to further understand these interactions and specific mechanisms.

Further limitations include the short-term follow-up period. Our study was designed to limit confounding factors that could influence the measure of treatment effectiveness of PN. We decided not to add new drugs or use other treatment methods to accurately evaluate pain. Although the participants’ compliance resulted in a dropout rate of only 9.8%, which is significantly lower than the expected dropout rate (20%), it would be very difficult to limit other treatment options for a longer period. Therefore, we had difficulties in executing a long-term follow-up study design.

Further research is needed that includes RCTs and long-term follow-up studies to verify the long-term efficacy of PN and related analyses based on AI algorithms are required. After this verification of the long-term effectiveness compared to control groups, the AI algorithm-based texture analysis method could be applied to a wider population of knee OA patients as a diagnostic tool. Moreover, if the long-term efficacy of the subchondral restoration of PN is proven, it could become a better choice for OA patients compared to other types of intra-articular injection materials in the early stage of ongoing subchondral remodeling.

## 5. Conclusions

In this study, we verified the functional improvements following PN-filler treatment in knee OA patients, as well as anatomical improvements in the microstructure of the subchondral bone layers. We detected a structural change after the administration of PN filler-treatment in knee OA patients through the automated AI algorithm-based texture analysis. Our study results imply that the AI algorithm-based texture analysis could be a promising tool for not only detecting knee OA progression and prognosis evaluation, but also for assessing treatment outcomes. Further studies, including RCTs and long-term follow-ups, should be conducted.

## Figures and Tables

**Table 1:** Demographic characteristics of the participants.

Variables	Male (*n* = 14)	Female (*n* = 37)	Total (*n* = 51)
Dropped out	1 (20%)	4 (80%)	5 (100%)
Sex	14 (27.5%)	37 (72.5%)	51 (100%)
Age (years)			
Mean ± SD	59.7 ± 9.7	63.9 ± 8.4	62.7 ± 8.9
Median (min–max)	58 (46–75)	65 (44–77)	64 (44–77)
Height (cm)			
Mean ± SD	167.5 ± 5.9	155.2 ± 6.1	158.7 ± 8.2
Median (min–max)	168 (156–176)	156 (143–165)	158 (143–176)
Weight (kg)			
Mean ± SD	71.1 ± 5.0	61.8 ± 8.8	64.4 ± 8.9
Median (min–max)	71.5 (60.3–71.7)	62.0 (46.0–72.1)	64.6 (46.0–72.1)
BMI (kg/m^2^)			
Mean ± SD	25.4 ± 2.7	25.7 ± 3.4	25.6 ± 3.2
Median (min–max)	58 (17.1–27.7)	65 (21.1–27.4)	25.6 (17.1–27.7)
Kellgren–Lawrence grade			
I	8 (15.7%)	20 (39.2%)	28 (54.9%)
II	5 (9.8%)	14 (27.5%)	19 (37.3%)
III	1 (2%)	3 (5.9%)	4 (7.8%)
Osteoarthritis site			
Medial	12 (23.5%)	30 (58.8%)	42 (82.4%)
Lateral	1 (2%)	4 (7.8%)	5 (9.8%)
General	1 (2%)	3 (5.9%)	4 (7.8%)
Coronal alignment			
Varus	9 (17.6%)	26 (51.0%)	35 (68.6%)
Valgus	1 (2%)	3 (5.9%)	4 (7.8%)
Neutral	4 (7.8%)	8 (15.7%)	12 (23.5%)
Injection side			
Right	9 (17.6%)	21 (41.2%)	24 (47.1%)
Left	5 (9.8%)	16 (31.4%)	27 (52.9%)

For continuous variables, the data are expressed as the mean ± standard deviation and median (minimal–maximal values). For categorical variables, the data are expressed as frequency and percentage. Osteoarthritis site was decided based on arthritis findings in knee X-rays.

**Table 2:** BSV changes between before and after PN injection of subgroups separated by depth and inner and outer layers.

	Before PN Injection		*p*-Value	After PN Injection		*p*-Value
	MC	LC		MC	LC	
SL	0.587 ± 0.029	0.553 ± 0.032	<0.001	0.568 ± 0.017	0.550 ± 0.017	0.0021
ML	0.555 ± 0.032	0.535 ± 0.033	<0.001	0.533 ± 0.018	0.535 ± 0.018	0.325
DL	0.552 ± 0.034	0.520 ± 0.033	<0.001	0.525 ± 0.019	0.518 ± 0.016	0.322

Mean BSV value of each layer and compartment is stated. Between-group comparison of BSV shows disappeared statistical significance after PN injection at ML and DL. The *p* values are based on the results of the one-way ANOVA comparing BSV of medial and lateral compartment. PN = polynucleotide; MC = medial compartment; LC = lateral compartment; SL = superficial layer; ML = middle layer; DL = deep layer; BSV = bone structure value. *p* < 0.05.

**Table 3:** Delta BSVs in each subgroup before and after PN injection.

	Before PN Injection	After PN Injection	
Layer	ΔB	ΔA	*p*
Superficial	0.015 (−0.430 to 0.400)	0.020 (−0.43 to 0.40)	0.0565
Middle	0.010 (−0.150 to 0.220)	0.000 (−0.190 to 0.190)	0.00078 *
Deep	0.020 (−0.160 to 0.210)	0.005 (−0.180 to 0.190)	0.00081 *

For continuous variables, the data are expressed as the median (minimal–maximal value). The *p*-values were adjusted using the Bonferroni multiple testing correction method after the Friedman test for comparison of the delta BSVs. BSV, bone structure value; PN, polynucleotide; ΔB, delta BSV before PN injection; ΔA, delta BSV after PN injection. * *p* < 0.05.

## Data Availability

The data presented in this study are available on request from the corresponding author.

## Appendix A

**Table A1:** Inclusion and exclusion criteria of this study.

Inclusion Criteria	Exclusion Criteria
(1) Male and female adults aged ≥40 and <80	(1) Intra-articular injection to the knee within 6 months prior to screening
(2) Ability to understand and comply with the study procedures and visit schedule	(2) History of a knee surgery
(3) Ability to understand and comply with the study procedures and visit schedule	(3) Chronic inflammatory diseases such as rheumatoid arthritis
(4) Diagnosis of arthritis according to the American College of Rheumatology (ACR) classification criteria at the screening visit	(4) Use of non-steroidal anti-inflammatory drugs (NSAIDs) within 48 h prior to screening
(5) Kellgren–Lawrence Grade of I–IV for the severity of arthritis based on a knee X-ray at the screening visit	(5) Use of immunosuppressants such as cyclosporine A or azathioprine within 6 weeks prior to screening
(6) Visual Analogue Scale (VAS) at rest score of ≥40 mm at the screening visit	(6) Orthopedic diseases that may affect or interfere with the therapeutic effect
(7) Women of childbearing potential who have agreed to use a contraception method throughout the study	(7) Habitual use of psychotropic or narcotic analgesics for ≥1 week within 8 weeks prior to screening
	(8) Psychological or psychiatric disorders that may affect a subject’s participation in the study
	(9) More severe pain in other areas of the body than in the knee bone and joint at the screening visit
	(10) Ligament instability of Grade II or higher upon physical examination at the screening visit (Grade 0 = no; Grade I = 0–5 mm; Grade II = 5–10 mm; Grade III ≥ 10 mm)
	(11) Participated in other intervention studies within 30 days prior to screening
